# Machine Learning-Based Prediction of the Adsorption Characteristics of Biochar from Waste Wood by Chemical Activation

**DOI:** 10.3390/ma17215359

**Published:** 2024-11-01

**Authors:** Jinman Chang, Jai-Young Lee

**Affiliations:** Department of Environmental Engineering, University of Seoul, 163 Seoulsiripdaero, Seoul 02504, Republic of Korea

**Keywords:** wood waste, biochar, adsorption properties, activated carbon, machine learning

## Abstract

This study employs machine learning models to predict the adsorption characteristics of biochar-activated carbon derived from waste wood. Activated carbon is a high-performance adsorbent utilized in various fields such as air purification, water treatment, energy production, and storage. However, its characteristics vary depending on the activation conditions or raw materials, making explaining or predicting them challenging using physicochemical or mathematical methods. Therefore, using machine learning techniques to determine the adsorption characteristics of activated carbon in advance will provide economic and time benefits for activated carbon production. Datasets, consisting of 108 points, were used to predict the adsorption characteristics of biochar-activated carbon derived from waste wood. The input variables were the activation conditions, and the iodine number of activated carbon was used as the output variable. The datasets were randomly split into 75% for training and 25% for model validation and normalized by the min-max function. Four models, including artificial neural networks, random forests, extreme gradient boosting, and support vector machines, were used to predict the adsorption properties of biochar-activated carbon. After optimization, the artificial neural network model was identified as the best model, with the highest coefficient determination (0.96) and the lowest mean squared error (0.004017). As a result of the SHAP analysis, activation time was the most crucial variable influencing the adsorption properties. The machine learning model precisely predicts the adsorption characteristics of biochar-activated carbon and can optimize the activated carbon production process.

## 1. Introduction

Activated carbon has excellent adsorption properties due to its high carbon content and specific surface area. For this high adsorption property, activated carbon is used in various fields of modern society, such as water purification, air purification, energy production, and storage. Along with the development of modern society and industrial fields, as water and air environmental pollution increase nationwide, the demand for activated carbon to treat it is also naturally increasing [1,2,3,4]. In addition, the demand for activated carbon is expected to increase further as environmental regulations such as emission allowance standards for domestic air pollutant discharge facilities and emission water quality standards for sewage treatment facilities are strengthened. The activated carbon global market increased from $3.17 billion in 2017 to $5.01 billion in 2024 and is expected to grow to $1.21 billion in 2034 [5,6]. However, the manufacturing process of activated carbon is rather complicated, including the carbonization and activation processes, which disadvantages the price of activated carbon [5,6,7].

One of the methods to solve the supply and demand problem of activated carbon is the production of activated carbon using waste resources [8]. Generally, the raw materials for activated carbon are mainly charcoal-based raw materials such as wood and coconut shells, and coal-based raw materials such as lignite or coal. Waste wood is generated from various sources such as forestry, sawmills, the wood industry, and the production industry during construction and demolition. Waste wood is a biomass type with high utilization, but it is mainly treated through landfill or incineration [9]. These treatments emit carbon dioxide and other pollutants, and therefore, recycling waste wood is important for reducing carbon dioxide emissions and preserving resources [10,11]. Waste wood consists of cellulose, hemicellulose, and lignin, which contains a large number of carbon components and has various functional groups such as carboxyl, hydroxyl, methoxy, and phenol groups, which are advantageous for the adsorption of pollutants, including heavy metals. The characteristics of biochar and biochar-activated carbon depend on the characteristics of the raw material, and these various functional groups and high carbon content can positively affect the characteristics of biochar and biochar-activated carbon [7,12,13,14].

Biochar-activated carbon is produced in two stages: carbonization and activation. During the carbonization stage, the raw material loses volatile matter through combustion or pyrolysis, increasing its carbon content. However, the carbonization stage alone does not create sufficient pores, so an activation stage is necessary to develop a porous structure. The activation process is divided into physical activation and chemical activation. Physical activation involves the oxidation reaction of carbon on the particle surface to develop a micropore structure using agents such as H_2_O and CO_2_. Chemical activation, on the other hand, uses chemicals like KOH and NaOH to expand the porous structure [15,16,17]. Waste wood contains many lignocellulose and primarily secures a porous structure through chemical activation.

However, biochar-activated carbon exhibits varying adsorption performance depending on its specific surface area and the size and distribution of its pores. These characteristics of biochar-activated carbon change according to the activation method, the chemicals used, temperature, and time. Additionally, the reaction between raw materials and reagents or the process of pore formation is complicated to explain physically, chemically, or mathematically, making it challenging to predict the adsorption properties of biochar-activated carbon. Therefore, if the adsorption properties of biochar-activated carbon can be determined in advance, it is expected to provide economic and time advantages in the production process of activated carbon.

Research utilizing machine learning techniques has recently been conducted to address the difficulties of analyzing data with such nonlinear relationships [18]. Machine learning, an artificial intelligence (AI) technique, refers to computer algorithms that solve problems by inferring relationships between data. These machine learning techniques are helpful for inferring nonlinear relationships between factors. They are applied in various fields of modern society with diverse and complex relationships, such as engineering, medicine, and finance [19,20]. Thus, applying machine learning to predict the adsorption performance of biochar-activated carbon will allow us to elucidate the relationship between variables and more accurately predict adsorption properties. Jiang et al. (2019) used linear regression, support vector regression, and a random forest model to predict the adsorption properties of activated carbon produced by hydrocarbon combined with pyrolysis. They demonstrated that the random forest model predicts adsorption properties well [21]. Wang et al. (2023) confirmed the feasibility of using machine learning models to predict the adsorption properties of activated carbon by predicting the BET-specific surface area and yield of activated carbon manufactured from various biomass using random forest models [22]. However, in these studies, raw materials’ activation conditions and physicochemical properties were used simultaneously to predict adsorption properties. Adding these variables leads to limitations, as the model is only sometimes used and relies on that data.

This study aims to predict the adsorption properties of biochar-activated carbon derived from waste wood under various activation conditions using machine learning models, including artificial neural networks, random forests, extreme gradient boost, and support vector machines, and comparing traditional linear regression methods. In addition, the study aims to compare the performance of these models to identify the most suitable model and to evaluate important factors such as types and proportions of reagents through SHAP analysis.

## 2. Materials and Methods

### 2.1. Production and Analysis of Biochar-Activated Carbon

The wood waste used in the study was domestic wood waste generated in S city. Biochar-activated carbon was produced by carbonizing waste wood using the hydrothermal carbonization method (HTC), and chemical activation was performed using KOH, ZnCl_2_, and NaOH. HTC is a technology that generates biochar by mixing raw materials and water and pyrolyzing the hydrocarbon chain of cellulose with water vapor pressure produced when vaporizing at 150 to 300 °C below the critical temperature of water [23,24]. Carbonization was performed at 300 °C for 4 h; the activation temperature, time, and the ratio of the reagents were carefully selected and are shown in Table 1. The weight ratios of the reagents to the carbonized material were set at 0.5, 1, and 1.5. The activation temperatures were set to 600 °C, 700 °C, 750 °C, and 800 °C, and the activation times were set to 30, 60, and 90 min. These temperatures were used primarily as the activated carbon manufacturing temperature for wood-based raw materials, producing biochar-activated carbon under various activation conditions [25,26,27,28]. The adsorption characteristics of the produced biochar-activated carbon were then analyzed by ASTM D 4607 for iodine number, a key factor in determining the quality of activated carbon and its adsorption performance [29].

### 2.2. Data Collection and Preprocessing

Experimental results were collected as the raw dataset to predict the adsorption properties of biochar-activated carbon, and the total number of datasets consists of 108 points. The input variables of the model were the type of reagent, the input rate, the activation temperature, and the activation time. Among the input variables, the types of reagents belonging to categorical data were preprocessed as numerical data for models to use as training materials using the one-hot encoding technique. The iodine number was used as the output variable. Iodine number is one factor that determines the quality of activated carbon and can evaluate the adsorption performance of activated carbon. For training and validation of the machine learning model, the 108 datasets were randomly split into 75% and 25%, and the data values were normalized to have values between 0 and 1 through the min-max function so that the machine learning model could learn on a common scale. A Pearson correlation analysis was performed to determine the correlation between the variables. Pearson correlation analysis is a technique that statistically analyzes the correlation between two variables and has a value between −1~1. The closer the correlation coefficient between variables is 1 or −1, the stronger the correlation, and the closer to 0, the more there is no relationship. Pearson correlation analysis was performed using the Seaborn library in a Python 3.9.19 environment.

### 2.3. Model Optimization and Evaluation

In this study, four models were developed, and the predictive performance was compared with traditional linear regression to predict the adsorption properties of bio-char-activated carbon. The models used are the artificial neural network (ANN), random forest (RF), extreme gradient boost (XGB), and support vector machine (SVM). Table 2 shows the primary hyperparameters and optimization ranges of each model. Hyperparameters refer to variables users set that are essential for the model’s construction, such as structure and number of iterations. Optimizing hyperparameters can improve the predictive performance of machine learning models. Hyperparameters and adjustment ranges were set considering the number of data in the commonly used range. The machine learning models were built using Python 3.9.19, and the model optimization was carried out through the Bayesian optimization technique. Considering the insufficient data points, K-fold cross-validation was used to evaluate the model. K-fold cross-validation is a way to ensure the reliability of the model with low available data by dividing the data into K segments and creating K models, training them in K-1 segments, and evaluating them in the remaining segments to average each validation index [30,31].

The performance of the models was evaluated based on the mean square error (MSE) and coefficient of determination ($R^{2}$) for the test data. The coefficient of determination means the fitness between the measured and predicted values, 0 to 1. As the coefficient of determination approaches 1, the model’s performance is more accurate. MSE is a value obtained by averaging the sum of squares of the errors of the experimental value and the predicted value, and a smaller value of MSE means that the smaller the error between the predicted value of the model and the actual measured value and the higher the accuracy of the model. The mean square error and coefficient of determination are calculated through Equations (1) and (2) [32,33].

(1) $$MSE=\frac{\sum_{i=1}^{N}{\left(y_{i}-\hat{y_{i}}\right)}^{2}}{N}$$

(2) $$R^{2}=1-\frac{\sum_{i=1}^{N}{\left(\hat{y_{i}}-y_{i}\right)}^{2}}{\sum_{i=1}^{N}{\left(\hat{y_{i}}-{\bar{y}}_{i}\right)}^{2}}$$

Here, N was the total number of data, $Y_{i}$ and ${\bar{Y}}_{i}$ were experimental values, the average values of the experimental values, and $\hat{Y_{i}}$ were the predicted values.

### 2.4. Model Selection and Interpretation

The adsorption properties of biochar-activated carbon are affected by various factors, and the degree of influence varies depending on each factor. Shapley Additive exPlanations (SHAP) analysis was performed to analyze the contribution between variables. SHAP is a method that applies the concept of Shapley value in game theory to machine learning to analyze the contribution of input variables, which makes it easy to interpret machine learning models, understand prediction logic, and verify the reliability and fairness of the model. The SHAP analysis was performed using the SHAP library in Python 3.9.19 [34].

## 3. Results and Discussion

### 3.1. The Adsorption Properties of Biochar-Activated Carbon

**Figure 1:**
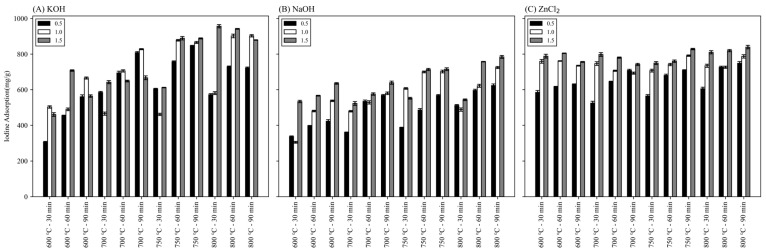
Iodine number of biochar-activated carbon in this study.

Iodine number is a crucial factor that determines the quality of activated carbon and is a measure of adsorption performance. Figure 1 shows the iodine number of biochar-activated carbon prepared by three reagents. All three reagents tended to increase the iodine number as the activation time increased at the temperatures of 600 °C, 700 °C, 750 °C, and 800 °C. At the same time, the iodine number tended to increase as the activation temperature increased. The increase in adsorption properties of biochar-activated carbon with increasing temperature is consistent with the well-known facts by prior research [35,36,37,38]. In addition, as the ratio of the reagent increased, the iodine number tended to increase. Biochar activated with KOH and NaOH shows the highest iodine number at 800 °C when the input ratio of the reagent is 1.5, which is judged to increase the specific surface area by forming micropores of activated carbon through carbon consumption in the activation process by KOH and NaOH. The gasification reaction was promoted as the amount of the reagent increased [36]. When the input ratio of the reagent is 1.5, the iodine number of biochar-activated carbon activated with ZnCl_2_ varies according to temperature. However, the effect seems insufficient, and when the input ratio of the reagent is small, the iodine number increases significantly due to the increase in temperature and time.

### 3.2. Data Analysis and Pre-Processing

**Figure 2:**
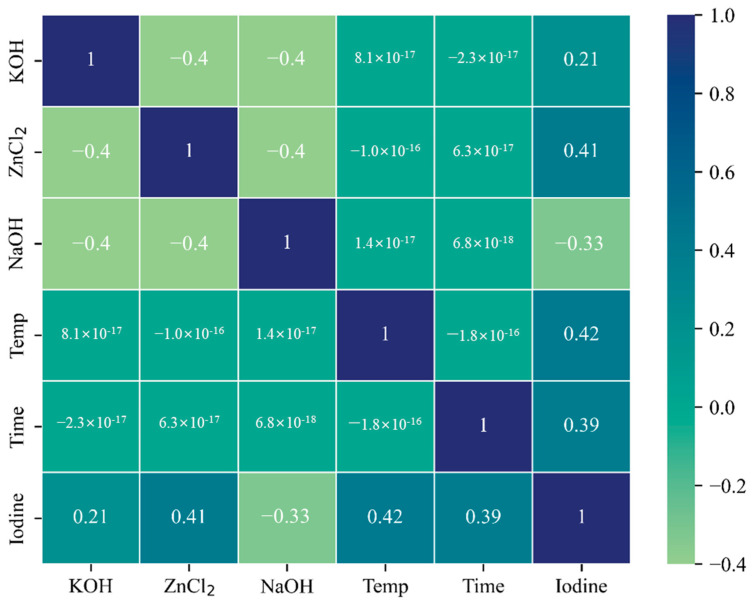
Pearson correlation matrix between variables.

A total of 108 data sets were constructed through the experiment, consisting of 5 activation conditions and biochar-activated carbon’s iodine number. Figure 2 shows the Pearson correlation coefficient between the variables used in the study. The correlation coefficient between the variables is −0.4 to 0.42. The correlation coefficient between the activation temperature and iodine number was 0.42, which was the largest, and the four activation conditions, excluding the NaOH reagent, showed a positive correlation. Among the reagents, ZnCl_2_ has the highest correlation with the iodine number, which is consistent with the study’s results. When chemically activated with various reagents, activated carbon activated with ZnCl_2_ showed high adsorption characteristics [37,38,39,40]. As a result of the Pearson correlation analysis, the activation temperature and activation time are the main factors affecting the adsorption characteristics of biochar-activated carbon.

### 3.3. Performance of Optimized Models

**Figure 3:**
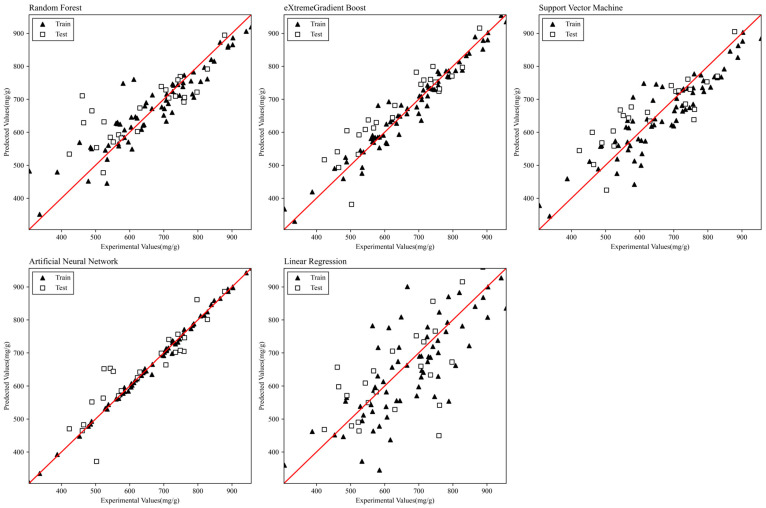
Scatter plots of predicted and experimental values for each model.

The performance of the four models was compared after applying the optimized hyperparameters shown in Table 3. Table 4 shows the MSE and $R^{2}$ values in the training and verification process of the four models and the linear regression model. In the training process, each model showed an MSE of 0.00007 to 0.00666, a coefficient of determination of 0.94 to 0.99, and higher performance than the linear regression model. In the test process, the MSE of 0.00402 to 0.00909 and the coefficient of determination of 0.90 to 0.96 were shown, and performance was higher than that of the linear regression model, the same as in the training process. This means the machine learning model is more suitable than the conventional linear regression model for learning nonlinear relationships between data. The four models showed high performance in predicting the adsorption characteristics of biochar-activated carbon, and the ANN model showed the best predictive performance. Figure 3 is a scatterplot showing the relationship between the experimental and predicted values. The closer the data is to the solid red line (y = x) in the scatterplot, the closer the predicted value is to the experimental value. The ANN model performed better than other models in training and verification processes. These results demonstrate the benefits of neural network-based models for regression problems and the potential of deep learning models. The ANN model has shown effective performance because it infers complex nonlinear relationships between properties and influencing factors of biochar-activated carbon [30,31,41]. The XGB model is the second most predictive model after ANN, and the data during the training process is well located on the diagonal of the graph. However, the data during the verification process have some variations, which is consistent with the fact that the MSE and R^2^ values of the XGB model are lower than those of the ANN model.

### 3.4. Variable Importance Analysis

**Figure 4:**
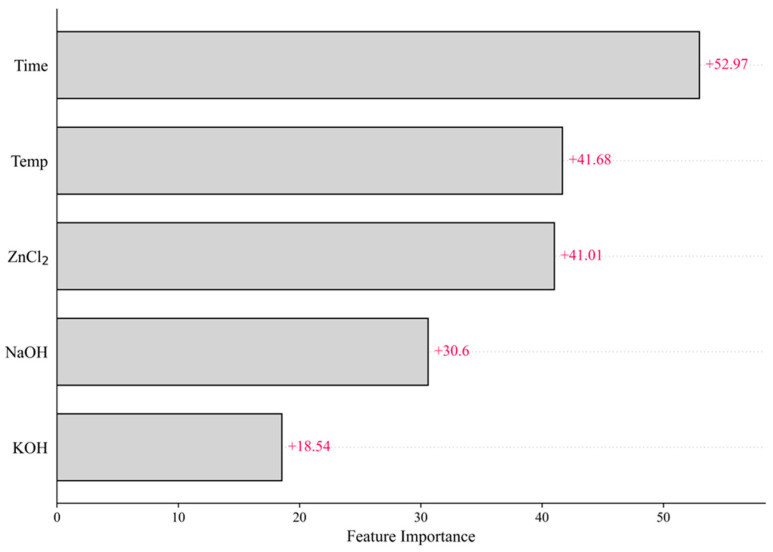
Effects of input variables on iodine adsorption of biochar-activated carbon.

Based on the ANN model, which showed the highest prediction accuracy in this study, a SHAP analysis was performed to analyze the importance of variables. The input variables were set to activation conditions, including temperature and time. Figure 4 is a graph showing the effect of each variable on the overall model through the sum of the absolute mean of each variable’s SHAP values. The longer the graph, the greater the impact on the model, and it can be seen as an essential variable in the prediction of the model. The SHAP analysis, in which activation time is the essential variable, differs from the results of the Pearson correlation, in which the activation temperature condition was the primary variable, in that the Pearson correlation only identified a linear correlation between the two variables. In contrast, the SHAP analysis considered the contribution of each variable to the machine learning model’s predictive ability [42].

**Figure 5:**
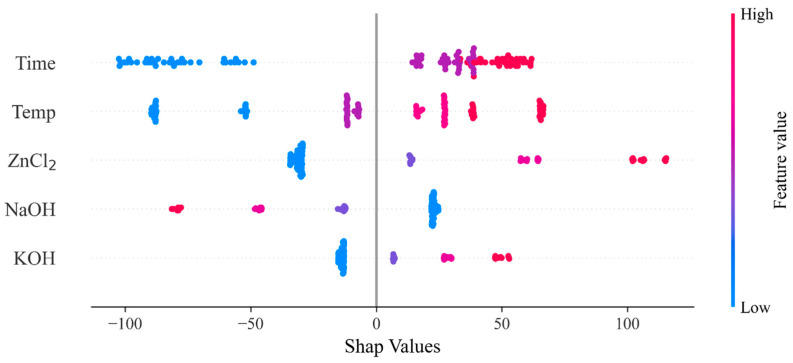
The result of the SHAP analysis.

**Figure 6:**
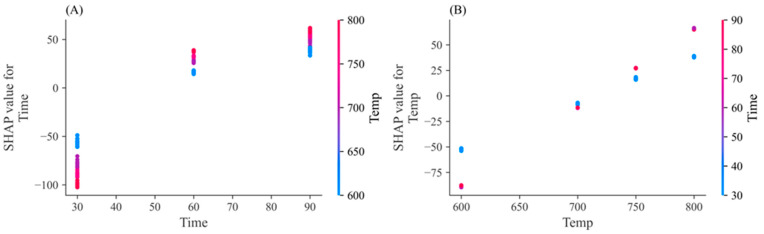
Dependence plot of temperature and retention time. (**A**) SHAP values of Temperature over Time, (**B**) SHAP values of Time over Temperature.

Figure 5 shows the contribution of each independent variable derived from the SHAP analysis. If the SHAP value is positive, it influences the increase in the predicted value. As the activation time and temperature increase, the SHAP value gradually increases and changes from negative to positive, which means it affects the increase in the predicted value as the activation temperature and time increase. Figure 6 shows the correlation between activation temperature and time. Activation temperatures below 700 °C have a negative effect on the forecast, while those above 700 °C have a positive effect on the forecasts. When the temperature is below 700 °C, the contribution decreases as the activation time increases; when the temperature exceeds 700 °C, the contribution increases as the activation time increases. As a result of the analysis of key factors, it was found that the main factors in the adsorption characteristics of biochar-activated carbon are activation temperature and activation time. The increase in temperature and the increase in reaction time promote the gasification reaction and the elution of minerals, causing a change in the adsorption properties of activated carbon in biochar. These results are consistent with previous studies in which the activation time is known to affect the adsorptive capacity of activated carbon, and it seems that when the activation time increases, the ash content of activated carbon increases, which interferes with pore formation and decreases micropores due to overactivation [21,22,36]. Therefore, to improve the performance of biochar-activated carbon in various environmental and energy fields, it is judged that an appropriate choice between activation temperature and activation time is required.

## 4. Conclusions

This study used a machine learning model to predict the adsorption properties of biochar-activated carbon. Four models were used: RF, XGB, SVM, and ANN. Each model showed high prediction performance with an MSE of 0.004017–0.009092 and a coefficient of determination of 0.90–0.96 during the testing process. After comparing the statistics of the Train dataset with the Test dataset, the ANN model was found to be the most suitable model. According to the analysis of the main factors using SHAP, it was found that the factor that significantly influences the adsorption properties of biochar-activated carbon is the activation time, and the activation time interacts with the activation temperature and affects the properties of biochar-activated carbon. The accurate prediction of waste wood adsorption properties by ANN-based machine learning models is a significant achievement. However, it’s crucial to note that this success results from extensive data and learning about diverse conditions and raw materials. This underscores the importance of conducting experiments and analyses in a wide range of conditions and environments to ensure the robustness of the model. When these conditions are met, machine learning models are expected to be able to effectively optimize the generation process of activated carbon utilizing waste in the future.

## Figures and Tables

**Table 1:** Activation conditions in this study.

Categories	Conditions
Agent of chemical activation	KOH, NaOH, ZnCl_2_
Ratio of chemical activation (agent/biochar)	0.5, 1, 1.5
Activation Temperature (°C)	600, 700, 750, 800
Activation Time (min)	30, 60, 90

**Table 2:** Variables for Machine Learning Models.

Model	Hyperparameter	Values
ANN	Hidden Layers	1, 2, 3
	Neurons	32, 64, 128, 256, 512
	Activation Function	identity, logistic, tanh, relu
	Solver	Lbfgs, sgd, adam
	Max Iteration	500, 1000, 1500, 2000
	Alpha (min, max, step)	0.0001, 1, 0.00001
	Learning Rate (min, max, step)	0.001, 1, 0.0001
RF	N_estimators (min, max, step)	10, 100, 1
	Max_depth (min, max, step)	2, 11, 1
	Min_samples_leaf (min, max, step)	2, 20, 1
	Min_samples_split (min, max, step)	2, 20, 1
	Max_features	sqrt, log_2_, None
SVM	C (min, max, step)	0.01, 10, 0.001
	Kernel	linear, poly, rbf
	Degree (min, max, step)	1, 10, 1
	Epsilon (min, max, step)	0.1, 0.5, 0.01
	Gamma	10^−4^, 10^−3^, 10^−2^, 0.1, 1, 10 10^2^, 10^3^, 10^4^
XGB	Max_depth (min, max, step)	3, 11, 1
	Learning_rate (min, max, step)	0.001, 1, 0.001
	Gamma	0, 10^−5^, 10^−4^, 10^−3^, 10^−2^, 0.1, 1
	Subsample (min, max, step)	0.1, 0.5, 0.05
	Colsample_bytree (min, max, step)	0.2, 1, 0.05
	Min_child_weight (min, max, step)	1, 10, 1
	N_estimators (min, max, step)	10, 100, 1

**Table 3:** Optimization of hyperparameters for machine learning models.

Model	Hyperparameter	Values	Model	Hyperparameter	Values
ANN	Hidden Layers	2	XGB	Max_depth	6
	Neurons	128 for each layer		Learning_rate	0.12
	Activation Function	relu		Gamma	10^−5^
	Solver	lbfgs		Subsample	0.45
	Max Iteration	2000		Colsample_bytree	0.85
	Alpha	0.00017		Min_child_weight	1.0
	Learning Rate	0.0039		N_estimators	71
RF	N_estimators	75	SVM	C	1.256
	Max_depth	11		Kernel	rbf
	Min_samples_leaf	2		Degree	4
	Min_samples_split	4		Epsilon	0.11
	Max_features	sqrt		Gamma	1

**Table 4:** MSE and R^2^ values of models in this study.

Model	Train		Test	
	MSE	$R^{2}$	MSE	$R^{2}$
LR	0.016273	0.85	0.017629	0.81
ANN	0.000065	0.99	0.004017	0.96
RF	0.003661	0.97	0.009092	0.90
SVM	0.006656	0.94	0.008833	0.90
XGB	0.000877	0.99	0.005415	0.94

## Data Availability

Dataset available on request from the authorities.
